# Surveillance of SARS-CoV-2 infection based on self-administered swabs, Denmark, May to July 2022: evaluation of a pilot study

**DOI:** 10.2807/1560-7917.ES.2023.28.38.2200907

**Published:** 2023-09-21

**Authors:** Kamille Fogh, Tine Graakjær Larsen, Cyril Jean-Marie Martel, Frederik Trier Møller, Lasse Skafte Vestergaard, Ramona Trebbien, Anne-Marie Vangsted, Tyra Grove Krause

**Affiliations:** 1 Statens Serum Institut, Copenhagen, Denmark

**Keywords:** Home-based testing system, COVID-19, surveillance, monitoring, PCR, WGS, self-test, self-swab

## Abstract

**Background:**

During the COVID-19 pandemic, the Danish National Institute for Infectious Disease, Statens Serum Institute (SSI) developed a home-based SARS-CoV-2 surveillance system.

**Aims:**

We wanted to determine whether a cohort of individuals performing self-administered swabs for PCR at home could support surveillance of SARS-CoV-2, including detection and assessment of new variants. We also aimed to evaluate the logistical setup.

**Methods:**

From May to July 2022, 10,000 blood donors were invited to participate, along with their household members. Participation required performing a self-swab for 4 consecutive weeks and answering symptom questionnaires via a web app. Swabs were sent by post to SSI for PCR analysis and whole genome sequencing. After study completion, participants were asked to complete a questionnaire concerning their experience.

**Results:**

In total, 2,186 individuals enrolled (47.4% blood donors), and 1,333 performed self-swabbing (53.0 blood donors), of whom 48 had at least one SARS-CoV-2-positive sample. Fourteen different Omicron subvariants, primarily BA.5 subvariants, were identified by whole genome sequencing (WGS). In total, 29 of the 63 SARS-CoV-2-positive samples were taken from individuals who were asymptomatic at the time of swabbing. Participants collected 2.9 swabs on average, with varying intervals between swabs. Transmission within households was observed in only three of 25 households.

**Conclusion:**

Participants successfully performed self-swabs and answered symptom questionnaires. Also, WGS analysis of samples was possible. The system can support surveillance of respiratory pathogens and also holds potential as a diagnostic tool, easing access to test for at-risk groups, while also reducing the burden on healthcare system resources.

**Key public health message**

**What did you want to address in this study?**

Throughout the COVID-19 pandemic, it has been necessary to monitor COVID-19 transmission patterns, even when SARS-CoV-2 testing capacity has been limited. We wanted to investigate whether home SARS-CoV-2 tests would provide information for surveillance and provide a different approach to monitoring the occurrence and evaluation of novel SARS-CoV-2 variants.

**What have we learnt from this study?**

We learned that the home-based surveillance system can be a feasible alternative to population testing in swab-testing centres and can be used for a wide array of pathogens. Participants can self-sample at home (including children) as well as register samples and symptoms using a web app. We were able to identify circulating SARS-CoV-2 variants, describe symptom patterns and study household transmission.

**What are the implications of your findings for public health?**

A home-based surveillance system can provide information about the prevalence of infection in the general population while using very few resources from the healthcare system. It offers information provided from tested individuals, e.g. self-reported symptoms, allowing for risk assessment of new pathogens or variants of concern. This system can help to define at-risk groups within the population, which may otherwise be hindered when testing activity is limited.

## Introduction

From the early phase of the COVID-19 pandemic, an important pillar in the Danish strategy for COVID-19 management was building up a high PCR test capacity to ensure easy and free access to severe acute respiratory syndrome coronavirus 2 (SARS-CoV-2) testing. Denmark has a population of ca 5.8 million people [1]. More than 66 million PCR tests were performed in Denmark from January 2020 to September 2022 [2], and more than a quarter of the Danish population was PCR-tested in some of the weeks during the first surge of the SARS-CoV-2 Omicron variant of concern (Phylogenetic Assignment of Named Global Outbreak (Pango) lineage designation B.1.1.529) [2]. On 1 February 2022, restrictions were lifted and recommendations for COVID-19 testing changed markedly and test activity slowed substantially [3].

On 1 April 2023, a complete cessation of publicly available testing was implemented, returning to a situation where PCR tests can only be requested by a doctor on clinical indication. In spite of this, the need to surveil COVID-19 prevalence in the general community remains important. Furthermore, in the event that a new SARS-CoV-2 variant of concern emerges, we must still have the capacity to test selected groups independently of health-seeking behaviours.

Statens Serum Institute (SSI) developed a pilot project for a home-based surveillance system to investigate whether a cohort of individuals performing self-swabbing for SARS-CoV-2 PCR analysis and answering symptom questionnaires can support or replace other existing surveillance systems. A test system of this kind has perspectives far beyond COVID-19 surveillance and can aid our preparedness for a future pandemic situation where rapid and efficient testing of the community is important. Such a system could enable surveillance of changing trends, viral load, duration of viral excretion and symptoms, as well as household transmission connected to the occurrence of new variants. The questionnaire could also be adapted to include exposures. With a sufficiently large cohort, the test system could be used to surveil the demographic spread of a microorganism, be it SARS-CoV-2 or another pathogen, and define risk groups, e.g. age groups, people in particular professions, or individuals with certain comorbidities, as well as infection routes, e.g. identify food or animal sources.

The objectives of the study were to (i) to assess if the system could be used to monitor trends, describe symptoms, detect household transmission and assess circulating variants of SARS-CoV-2 and (ii) evaluate the test system setup including participation rate and adherence and to identify improvement possibilities.

## Methods

### Study setting

During the study period of May to July 2022, the incidence of SARS-CoV-2 in the Danish population was between 56 and 274 per 100,000 inhabitants. Variants circulating were primarily Omicron BA.5 and its subvariants along with a smaller proportion of BA.4 and BA.2 (and subvariants of these) [4,5]. Testing was not recommended for the general population unless high age (≥ 65 years) or other risk factors could provide indication for early treatment. Denmark had fully vaccinated (primary schedule, 2 doses) 81% of the population and no public health recommendations of non-pharmaceutical interventions like face mask-wearing in public were in place [6].

### Study cohort

Because previous experience has shown that blood donors will participate at a higher rate than the general population [7], active blood donors affiliated with the Danish blood banks were chosen as the primary cohort for this pilot study. Blood donors’ ages range from 17 to 75 years, they have an evenly distributed sex composition, broad geographical representation and are healthier than the general population [8]. To reduce the bias of this selection, blood donors’ household members were also invited to enrol. It was therefore expected that the cohort would provide a reasonably broad sample of the Danish population.

There were no age limits for participants, and no exclusion criteria. Written information was provided for the participants and available in Danish. Participation was voluntary. The Danish Civil Registration number (CPR) register (a unique Danish Civil Registration system) was used to acquire home addresses of participating blood donors and identify individuals living in the same household. Also, the CPR register was used to identify persons with parental custody of participating children under 15 years of age.

### Study design

A total of 10,000 blood donors in Denmark were invited via a secure digital mailbox system (Digital Post) to participate in the pilot study. The invitations with a link to a registration form were sent out on 18 May 2022, and the study continued until 13 July 2022. Blood donors could register themselves as well as household members for the study using the registration form. 

Participation included self-sampling with a swab in the oropharynx and the anterior part of the nasal cavity, as this has been shown to provide the highest sensitivity and specificity [9]. Children under the age of 15 years were only required to swab in the nose. Swabs were to be performed once per week for 4 weeks. Detailed information about the project was provided with the invitation letter and on a project website, which included an instructional video on how to perform the swab correctly. It was possible for participants to contact project support by mail and telephone. 

After the study period ended, participants were sent a letter via Digital Post, thanking them for their participation, with a link to a questionnaire, which focused on their experience with participation in the study. Each participant over age 15 years received test results via the Danish national online laboratory response portal, where the test result is also available for healthcare workers. Parents of participants under age 15 years received test results for their children via Digital Post.

If a swab result was positive, participants were encouraged to have the result verified with a PCR test performed by trained personnel in a regular testing venue. Participants were encouraged to follow the Danish Health Authority's guidelines regarding positive SARS-CoV-2 test results. Regardless of test results, participants were encouraged to continue regular sampling each week for a total of 4 weeks.

### Sample collection and analysis

Test material including nylon flocked dry swabs, tongue spatulas, plastic sample tubes, alcohol wipes, transportation tubes, prepaid return envelopes and an instruction leaflet was sent to the home addresses of participating blood donors using regular mail. The test material was sent between 24 May to 1 June 2023. If the blood donor had signed up members of their household, the amount of test material sent matched the number of enrolled household members.

Samples were analysed at SSI, in the same way as all other samples from the Danish public testing system, with quantitative real-time RT-PCR (qRT-PCR) targeting the E-gene of the SARS-CoV-2 genome. The sample results were classified as ‘detected’ if the quantification cycle value was between 10 and 38, ‘inconclusive’ if it was between 38 and 40, and ‘not detected’ if it was between 0 and 10 as well as 40 and 45. Error-inconclusive (EI) results were attributed to samples that were registered incorrectly, e.g. if multiple CPR numbers were registered to the same sample tube, or if the samples had not arrived at SSI within 7 days of registration in the web app.

Whole genome sequencing (WGS) was performed at SSI on SARS-CoV-2-positive samples. Samples that failed WGS were reanalysed multiple times. The project samples were analysed in one batch along with other samples from the random sampling surveillance of all SARS-CoV-2-positive samples from the public testing system. A description of the WGS method can be found elsewhere [10,11].

### Data collection

Aggregated data from the Danish blood banks including age, sex and geographical information on invited blood donors were obtained.

Questionnaire data from the electronic database SurveyXact (SurveyXact, Denmark) were obtained from two different questionnaires: (i) The first was sent through a unique link provided in the digital invitation letter (available as Supplementary Questionnaire S1). Here, blood donors could register up to six participating household members, regardless of age. If a blood donor did not want to participate, they were asked to explain why not. These data were used to assess characteristics of this group as well as identify improvement possibilities; (ii) After the study period ended, the enrolled blood donors were sent a letter thanking them for their participation and asking them to access another questionnaire concerning their experience with participating, which they were asked to fill out on behalf of their household (available as Supplementary Questionnaire S3).

The participants used a web app specifically designed for the project to register personal samples and answer a third questionnaire (iii) about symptoms (available as Supplementary Questionnaire S2). Upon each sample registration, the web app provided a short instruction on how to complete the self-sampling. Samples were registered by using the web app to scan a data matrix code on the lid of the sampling tube. Login to the web app with a CPR number was required, thus ensuring that a personal sample was registered to an identifiable person. Participants were asked to send samples to SSI by regular, prepaid mail within 24 h of completing the self-swab.

### Statistical analyses

Participants were grouped by age and sex when relevant for analyses. Participants were also stratified according to geographical place of living, using their home address (from the CPR register). Households were identified using data from the CPR register: participants living on the same home address were defined as members of the same ‘household’. The presented data are primarily descriptive. When analysing the data, distinction was made between (i) the invited cohort, (ii) the enrolled cohort, which were those who signed up to participate and received test materials, and (iii) the tested cohort, from whom samples were received and analysed at SSI. All analyses of data and graphical representations were made using R version 4.1.3 [12].

## Results

**Figure 1:**
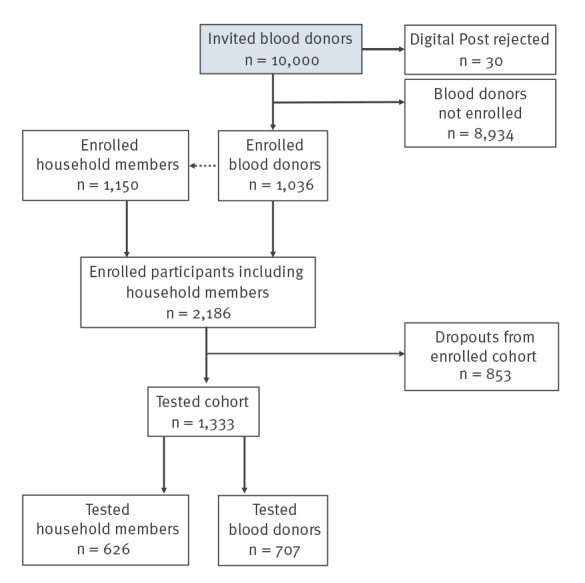
Flow chart of participants included in the study, Denmark, May–July 2022

Of the 10,000 blood donors who were sent an electronic invitation, 1,036 enrolled (10.4%) (Figure 1,Table 1). Blood donors registered on average 2.1 household members, including themselves, yielding an enrolled cohort size of 2,186 individuals. Statens Serum Institut received tests from 1,333 individuals from the enrolled cohort (61.0%), of whom 707 were among the originally invited blood donors (7.1% of the invited cohort). Supplementary Table S1 provides the demographic characteristics of these tested blood donors and the 626 household members who also performed tests and submitted samples. With a participation rate of only 1,333 people (who performed and sent self-administered swab tests to analysis), the study was not powered to assess changes in the prevalence of SARS-CoV-2.

**Table 1:** Demographic characteristics of the participants recruited for the study, Denmark, May–July 2022 (n = 10,000)

Characteristics	Blood donors invited		Blood donors enrolled		Tested of total enrolled participants (n = 2,186)		Positive of tested participants (n = 1,333)		Positive %^a^
	n	%	n	%	n	%	n	%	
Total	10,000	100	1,036	10.4	1,333	61.0	48	3.6	3.6
**Sex**									
Female	5,295	52.9	610	58.9	728	54.6	21	43.8	2.9
Male	4,705	47.0	426	41.1	605	45.4	27	56.2	4.5
**Age group (years)**									
0–4	0	0.0	0	0.0	14	1.1	0	0.0	0.0
5–9	0	0.0	0	0.0	49	3.7	0	0.0	0.0
10–14	0	0.0	0	0.0	87	6.5	3	6.2	3.5
15–19	203	2.0	5	0.5	61	4.6	1	2.1	1.6
20–29	2,441	24.4	138	13.3	157	11.8	5	10.4	3.2
30–39	1,864	18.6	131	12.6	118	8.9	3	6.2	2.5
40–49	2,155	21.6	216	20.8	215	16.1	13	27.1	6.0
50–59	2,148	21.5	309	29.8	349	26.2	16	33.3	4.6
60–69	1,151	11.5	226	21.8	255	19.1	7	14.6	2.7
70–79	38	0.4	11	1.1	27	2.0	0	0.0	0.0
≥ 80	0	0.0	0	0.0	1	0.1	0	0.0	0.0
**Residence**									
Central Region	2,351	23.5	271	26.2	336	25.2	14	29.2	4.2
Region Zealand	1,264	12.6	139	13.4	196	14.7	3	6.2	1.5
Capital Region	3,061	30.6	297	28.7	378	28.4	18	37.5	4.8
Region North	1,101	11.0	102	9.8	123	9.2	3	6.2	2.4
Region South	2,193	21.9	227	21.9	300	22.5	10	20.8	3.3

^a^ Percent positive of the total population that is tested, within group.

Most blood donors who did not enrol (n = 9,293) also did not answer the questionnaire concerning the reason(s) they did not participate (available as Supplementary Questionnaire S1). Of the 51 individuals who did answer, 22 answered that their reason was ‘not wanting to perform self-swabbing on themselves or their children’, 12 answered they ‘were going on vacation’ and nine answered ‘participation seems too time consuming’. Seven did not wish to participate, and one answered that they found the technology (i.e. smartphone app) too complicated.

A total of 219 enrolled blood donors answered ‘no’ to having collected all samples (in the questionnaire found as Supplementary Questionnaire S3). Most individuals (34.2%, n = 75) reported ‘malfunctions with the app’ as well as ‘forgetting to do it’ (16.4%, n = 36) and ‘being on vacation’ (15.5%, n = 34) as their reason. Few reported that ‘swabbing was difficult or uncomfortable’ (5.5%, n = 12).

During the study period, 3.6% of the tested population (n = 48) had a sample where SARS-CoV-2 was detected with PCR. The highest percentage of positive samples was among male participants (cf.d with female participants, p = 0.18) and among individuals in the age group 40–49 years (p = 0.07, when comparing with the other age groups as a whole).

### Samples and variant analysis

A total of 3,837 samples were received at SSI from 1,333 individuals, giving an average of 2.9 samples per participant. Of the tested cohort, 39% (n = 524) sent the four samples that were expected during the study period. Some participants (n = 21) completed and sent more than four samples. The interval between sample registrations ranged between 1 and 20 days. Most of the cohort performed consecutive self-sampling with an interval of 7 days. Supplementary Figure S1 provides a graphical representation of the sampling intervals during the study.

In 92% (3,539/3,837) of samples received, SARS-CoV-2 was not detected, while 23 samples were inconclusive and 63 samples from 48 individuals were found positive for the virus. For 212 samples, the analysis result ‘error-inconclusive’ (EI) was given. A total of 88 of these were never received at SSI. Twelve EI samples had been registered more than once, and therefore could not be safely connected to a specific participant. The last 112 (53%) EI samples were received at SSI, but after the 7-day threshold. One of these samples was received on day 25 after web app registration, though most samples (71/112) were received on day 8. The 112 received EI samples were analysed upon arrival to SSI, and in two of these (received on day 8 and day 10) SARS-CoV-2 was detected. Samples classified as EI came from all regions of Denmark with no apparent trend in geographical distribution.

The time from a sample was registered in the web app, and until samples were analysed was longer for EI samples (5.3 days) compared to samples where SARS-CoV-2 was detected/not detected in the samples (4.1 days).

### Participants with detected SARS-CoV-2

**Figure 2:**
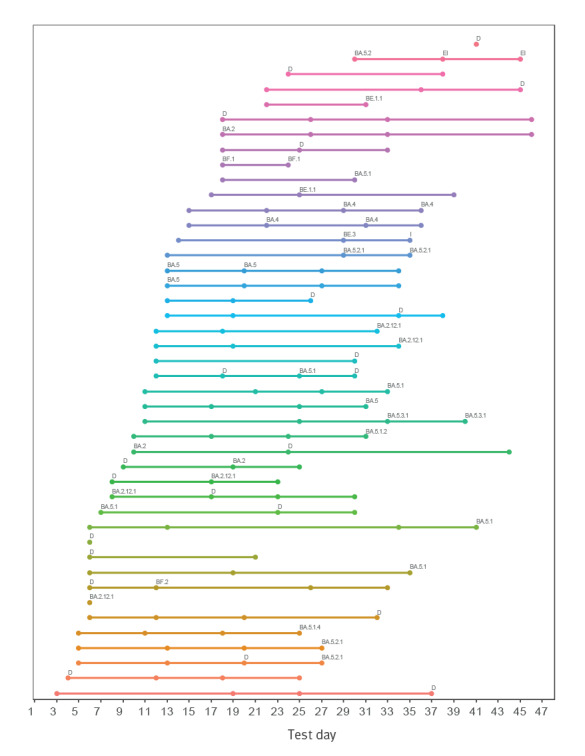
Testing series of participants who had at least one sample positive for SARS-CoV-2, Denmark, May–July 2022 (n = 48)

Figure 2 provides an overview of all individuals who had at least one sample positive for SARS-CoV-2. Participants started self-swabbing at different time points, performed a variable number of swabs and had varying intervals between consecutive samples. One participant had three positive consecutive samples, with 12 days between the first and third samples, while several other individuals had two samples with SARS-CoV-2 in a row. The longest period with continuous positive samples was 14 days and none of the participants exhibited viral rebound.

D: detected; SARS-CoV-2: severe acute respiratory syndrome coronavirus 2; WGS: whole genome sequencing.

Each line represents one person. Swabs are indicated by dots, and the test result on the swab is ‘not detected’ unless otherwise stated. If a SARS-CoV-2 variant was determined with WGS, this result is shown. If WGS was not successfully performed, D for ‘detected’ is shown.

### Symptoms reported by participants

**Figure 3:**
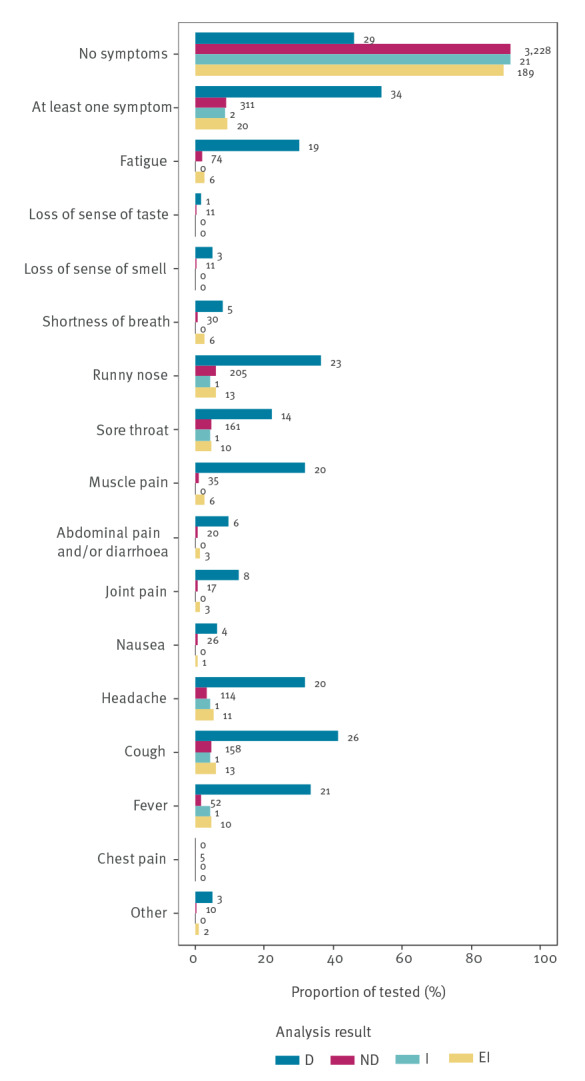
Symptoms reported at the time of swab collection, stratified by SARS-CoV-2 test result, Denmark, May–July 2022 (n = 3,837 samples)

More than 50% (34/63) of samples with detected SARS-CoV-2 came from individuals who reported at least one symptom, and 29 were asymptomatic (Figure 3). The most frequently reported symptoms among individuals with SARS-CoV-2 were cough, fever, runny nose, muscle soreness, fatigue and sore throat. Furthermore, 311 individuals without detected SARS-CoV-2 also reported symptoms. The questionnaire is available as Supplementary Questionnaire S2.

D: detected; EI: error-inconclusive; I: inconclusive; ND: not detected; SARS-CoV-2: severe acute respiratory syndrome coronavirus 2.

Numbers indicate the quantity, while bars represent the proportion, of sample results of participants who reported symptoms at the time of sampling.

### Whole genome sequencing

Figure 2 also includes results of WGS, which was attempted on all 63 positive samples. A variant was successfully determined in 38 of the samples (Table 2). The 25 (40%) samples that failed were on a WGS plate among other, routine WGS samples that also failed. Fourteen different variants were detected: subvariants of BA.5, especially BA.5.2.1 and BA.5.1 (including their subvariants), as well as BA.2.12.1. A few other BA.2 and BA.4 were also found. None of the participants were infected with more than one variant in the study period.

**Table 2:** Variant analysis results of SARS-CoV-2-positive samples, Denmark, May–July 2022 (n = 63)

Variants detected	Samples	%
Unknown	25	39.7
BA 5.1	6	9.5
BA 2.12.1	5	7.9
BA 5.2.1	5	7.9
BA 4	4	6.4
BA 5	4	6.4
BA 2	3	4.8
BA 5.3.1	2	3.2
BE 1.1	2	3.2
BF 1	2	3.2
BA 5.1.2	1	1.6
BA 5.1.4	1	1.6
BA 5.2	1	1.6
BE 3	1	1.6
BF 2	1	1.6
Total	63	100

SARS-CoV-2: severe acute respiratory syndrome coronavirus 2.

The variants detected show 14 different SARS-CoV-2 Omicron variants in 38 samples, and the 25 samples classified as unknown failed in sequencing.

### SARS-CoV-2 detection in households

**Figure 4:**
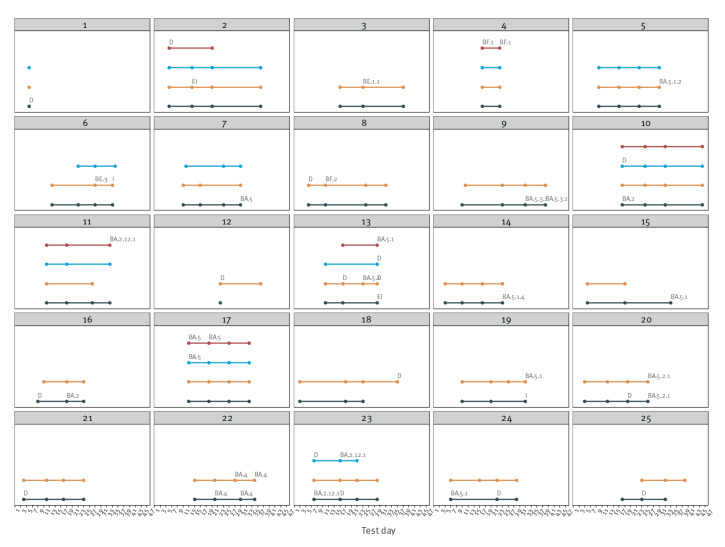
Test results and variant analysis for households^a^ with at least one positive SARS-CoV-2 test, Denmark, May–July 2022 (n = 25)

Figure 4 shows the 25 identified households where at least one of the participating household members had a sample with detected SARS-CoV-2. Six of these households included more than one person with detected SARS-CoV-2, and three of these showed signs of transmission within the household. In only nine of the households had all participants collected and sent four samples each.

D: detected; EI: error-inconclusive; I: inconclusive.

^a^ Households had two or more members.

Each row in each household represents the swab testing series for one person. Swabs are indicated by dots, and the test result on the swab is ‘not detected’ unless otherwise stated. If a variant was determined with WGS, this result is shown. If WGS was not successfully performed, D for ‘detected’ is shown.

## Discussion

Over a 4-week period, participants were successful in performing self-swabbing at home and providing information about symptoms via a specialised web app. The web app was useful for sample registration and questionnaire management. Whole genome sequencing analysis of the samples was possible on some, but not all samples, and household transmission was observed. With a sufficiently powered sample size, the system would also be able to detect changes in SARS-CoV-2 prevalence.

Geographical representation was achieved in the enrolled cohort while females and age groups between 40 and 69 years were overrepresented. This tendency has been observed previously in questionnaire-based cohort studies [13-15]. While 10.4% of the invited blood donor cohort enrolled in the study, only 7.1% of the invited cohort participated actively. Also, 39% of enrolled participants did not participate even though test material was sent to them. No incentives were provided, which could have affected the participation rate negatively. Geographical differences were observed in the tested cohort, which may reflect challenges in returning samples to SSI via mail for individuals living in more rural areas of Denmark. The largest number of participants came from the capital region. To achieve a higher participation rate, a different logistical setup could be used, e.g. participants could retrieve test material and send samples via pharmacies, convenience stores or their employer. This may also decrease the response time and the number of EI results. The threshold of 7 days for automatic EI response should be reevaluated, since many of the samples arrived at SSI in the following days.

SARS-CoV-2 was detected in only 48 individuals in our cohort during the study. The most frequently detected variants were BA.5 subvariants, and the most common variants in the cohort were also among the most common variants found in the general public in the same period [4,5]. Almost half of the SARS-CoV-2-positive individuals were asymptomatic at the time of testing. Denmark has a high SARS-CoV-2 vaccination coverage, so it can be assumed that most of the adult cohort was vaccinated. In addition, many Danish residents had had a previous BA.2 infection [16]. Our results indicate that in a population with a high level of hybrid immunity, the variants circulating during the study period may have led to asymptomatic infection in nearly half of those infected. Symptoms of BA.5 described in other studies are similar to our findings [17].

Home-based self-sampling studies have been performed previously and some have actively been used in the surveillance of COVID-19, e.g. the ‘COVID-19 infection survey’ [18] and the REACT-1 study in the United Kingdom [19]. Both of these large surveillance systems provided incentives for the participants, and correspondingly participation rate was high, e.g. 51% of households that were invited in England in the initial invitations enrolled in the COVID-19 infection survey [20] and 41% of individuals invited in the base sample of the REACT-1 study registered [21]. Other similar studies without incentives have been performed previously, e.g. the ‘Swede-I’ study from Sweden [22] and the ‘GoViral’ study, Massachusetts, United States [23]. These studies, like ours, had low (16% and 20%) participation rates. In Denmark, only a few studies of self-performed tests at home have been conducted previously, e.g. the project ‘Testing Denmark’ with more than 1.2 million Danes invited and 24.5% providing a sample [13] and the randomised controlled trial ‘DANMASK-19’, with a participation rate at 80% [24]. Both studies were conducted at an earlier stage of the pandemic, which may explain why participants were more willing to participate, despite the absence of incentives. 

A home-based self-sampling system like that presented here would make it easier for citizens to obtain SARS-CoV-2 tests. The setup could be used for screening activities in cases of outbreaks of new variants of concern. Thereby, the system could limit both costs and resources otherwise associated with testing for SARS-CoV-2 in the healthcare sector. In addition, the test system could be used for diagnosis and surveillance of other microorganisms.

A key strength of this surveillance system is the availability of anamnestic information from participants. There is, however, the possibility that the individuals are pre- or post-symptomatic at the time of sample registration. Also, a weekly symptom questionnaire provides only a static description of symptoms, while they are dynamic by nature. The system can, however, be changed in design to overcome challenges of this kind. For example, participants could be asked to perform daily samples after an initial positive sample or whenever symptoms are present.

We cannot be certain that individuals registering more than four samples in fact collected all the samples from themselves. They could instead mistakenly have registered their household members’ samples as their own on the web app. Future versions of this system should preclude a sample tube from being scanned more than once, and for individuals to be able to register more than one sample at a time. Also, the cohort’s behaviour varied regarding sampling intervals and number of samples collected per participant. Sending a reminder via the web app (e.g. a push notification) could increase adherence to performing all weekly swabs in a timely manner.

A key limitation to this pilot study is a low participation rate among invitees. The pilot project was carried out during a period impacted by both public holidays and summer vacation, which has likely influenced the participation rate. Blood donors have been widely used in several SARS-CoV-2-related projects in Denmark, and dropouts or lack of interest in participating may be on account of prior participation in other SARS-CoV-2-related studies. The study was conducted after major SARS-CoV-2 waves, most of the population had already been vaccinated and/or infected and restrictions had been lifted. Furthermore, there was a general ‘COVID-19 fatigue’ in the population at the time of the study, as evidenced by low overall testing activity and a low rate of infection in the society over the period. We were able to observe transmission within three households, and no households showed signs of more than one introduction of SARS-CoV-2. However, it is likely that transmission also could have occurred within the households before and/or after the study period. Another limitation encountered was related to WGS analysis of all samples on the same plate. This made the study vulnerable to laboratory contamination, which resulted in almost 40% of positive samples failing WGS. Because of this outcome, we cannot conclude with certainty that all study samples were of a good enough quality to be sequenced. The interval between swabs varied, and less than half of the cohort performed all four swabs. We assume that all participants received their test material, but it cannot be ruled out that some of the 39% enrolled individuals, who never performed swabs, did not receive their test material.

## Conclusion

Participation rate in this study was low. However, participants completed self-swabs and symptom questionnaires successfully. Whole genome sequencing analysis of the received samples was successful. The system can support respiratory pathogen surveillance and has the potential to be a diagnostic tool, easing access to test for relevant at-risk groups while also decreasing the burden on healthcare system resources.

## Supplementary Data

236000 Supplement

## References

[1] Denmark S. Population figures. Copenhagen: Statistics Denmark; 2022. Available from: https://www.dst.dk/en/Statistik/emner/borgere/befolkning/befolkningstal

[2] Statens Serum Institut (SSI). COVID-19 dashboard. Copenhagen: SSI; 2022. Available from: https://experience.arcgis.com/experience/aa41b29149f24e20a4007a0c4e13db1d/page/Nationalt

[3] Gram MA Steenhard N Cohen AS Vangsted AM Mølbak K Jensen TG Patterns of testing in the extensive Danish national SARS-CoV-2 test set-up. PLoS One. 2023;18(7):e0281972. 10.1371/journal.pone.0281972 37490451 PMC10368237

[4] Statens Serum Institut (SSI). Ugentlige tendenser: covid-19 og andre luftvejsinfektioner: uge 27 2022. [Weekly trends: COVID-19 and other respiratory infections: week 27 2022]. Copenhagen: SSI; 2022. Danish. Available from: https://files.ssi.dk/covid19/tendensrapport/rapport/ugentlige-tendenser-covid19-andre-luftvejs-uge27-2022-42jk

[5] Statens Serum Institut (SSI). Ugentlige tendenser: covid-19 og andre luftvejsinfektioner: uge 28 2022. [Weekly trends: COVID-19 and other respiratory infections: week 28 2022]. Copenhagen: SSI; 2022. Danish. Available from: https://files.ssi.dk/covid19/tendensrapport/rapport/ugentlige-tendenser-covid19-andre-luftvejs-uge28-2022_fdj1

[6] Mathieu E, Ritchie H, Rodés-Guirao L, Appel C, Giattino C, Hassell J, Macdonald B, Dattani S, Beltekian D, Ortiz-Ospina E and Roser M. Coronavirus Pandemic (COVID-19). OurWorldInData.org: 2020. Available from: https://ourworldindata.org/coronavirus

[7] Kaspersen KA Hindhede L Boldsen JK Mikkelsen S Vestergaard LS Berthelsen AN Estimation of SARS-CoV-2 infection fatality rate by age and comorbidity status using antibody screening of blood donors during the COVID-19 epidemic in Denmark. J Infect Dis. 2022;225(2):219-28. 10.1093/infdis/jiab566 34788834 PMC8689980

[8] Brodersen T Rostgaard K Lau CJ Juel K Erikstrup C Nielsen KR The healthy donor effect and survey participation, becoming a donor and donor career. Transfusion. 2023;63(1):143-55. 10.1111/trf.17190 36479702 PMC10107247

[9] Hørlyck S Nielsen SH Gress T Schneider U Martel CJ Steenhard N Combined nasal- and oropharyngeal self-swab provides equivalent performance compared to professionally collected oropharyngeal swabs in detecting SARS-CoV-2 in a real-life setting. J Virol Methods. 2023;313:114667. 10.1016/j.jviromet.2022.114667 36572155 PMC9783189

[10] Lyngse FP Kirkeby CT Denwood M Christiansen LE Mølbak K Møller CH Household transmission of SARS-CoV-2 Omicron variant of concern subvariants BA.1 and BA.2 in Denmark. Nat Commun. 2022;13(1):5760. 10.1038/s41467-022-33498-0 36180438 PMC9524324

[11] Hansen CH Friis NU Bager P Stegger M Fonager J Fomsgaard A Risk of reinfection, vaccine protection, and severity of infection with the BA.5 omicron subvariant: a nation-wide population-based study in Denmark. Lancet Infect Dis. 2023; 23(2):167-76. 10.1016/S1473-3099(22)00595-3 36270311 PMC9578720

[12] R Core Team. R: A language and environment for statistical computing. Vienna: R Foundation for Statistical Computing; 2020. Available from: https://www.R-project.org

[13] Fogh K Strange JE Scharff BFSS Eriksen ARR Hasselbalch RB Bundgaard H Testing Denmark: a Danish nationwide surveillance study of COVID-19. Microbiol Spectr. 2021;9(3):e0133021. 10.1128/Spectrum.01330-21 34908473 PMC8672904

[14] Sørensen AIV Spiliopoulos L Bager P Nielsen NM Hansen JV Koch A A nationwide questionnaire study of post-acute symptoms and health problems after SARS-CoV-2 infection in Denmark. Nat Commun. 2022;13(1):4213. 10.1038/s41467-022-31897-x 35864108 PMC9302226

[15] Fogh K Eriksen ARR Hasselbalch RB Kristensen ES Bundgaard H Nielsen SD Seroprevalence of SARS-CoV-2 antibodies in social housing areas in Denmark. BMC Infect Dis. 2022;22(1):143. 10.1186/s12879-022-07102-1 35144550 PMC8830972

[16] Statens Serum Institut (SSI). Seroprævalensundersøgelse af bloddonorer – 3. runde. [Seroprevalence study of blood donors – 3rd round]. Copenhagen: SSI; 2022. Danish. Available from: https://covid19.ssi.dk/-/media/arkiv/subsites/covid19/overvaagningsdata/moerketal/seropraevalensundersoegelse_runde3_version2.pdf?la=da

[17] Goller KV Moritz J Ziemann J Kohler C Becker K Hübner NO Differences in clinical presentations of Omicron infections with the lineages BA.2 and BA.5 in Mecklenburg-Western Pomerania, Germany, between April and July 2022. Viruses. 2022;14(9):2033. 10.3390/v14092033 36146837 PMC9506148

[18] Walker AS Vihta KD Gethings O Pritchard E Jones J House T Tracking the emergence of SARS-CoV-2 Alpha variant in the United Kingdom. N Engl J Med. 2021;385(27):2582-5. 10.1056/NEJMc2103227 34879193 PMC8693687

[19] Imperial College London (ICT). Real-time Assessment of Community Transmission (REACT) Study. London: ICT; 2022. Available from: https://www.imperial.ac.uk/medicine/research-and-impact/groups/react-study

[20] Office for National Statistics. Coronavirus (COVID-19) Infection Survey: technical data. 2020 edition of this dataset. London: ons.gov.uk. [Accessed: 07 Sep 2023]. Available from: https://www.ons.gov.uk/peoplepopulationandcommunity/healthandsocialcare/conditionsanddiseases/datasets/covid19infectionsurveytechnicaldata

[21] REACT Study Investigators; Riley S, Ainslie KEC, Eales O, Jeffrey B, Walters CE, et al. Community prevalence of SARS-CoV-2 virus in England during May 2020: REACT study. medRxiv 2020.07.10.20150524. Preprint. 10.1101/2020.07.10.20150524

[22] Plymoth A Rotzen-Ostlund M Zweygberg-Wirgart B Sundin CG Ploner A Nyren O Self-sampling for analysis of respiratory viruses in a large-scale epidemiological study in Sweden. Euro Surveill. 2015;20(11):21063. 10.2807/1560-7917.ES2015.20.11.21063 25811646

[23] Goff J Rowe A Brownstein JS Chunara R. Surveillance of acute respiratory infections using community-submitted symptoms and specimens for molecular diagnostic testing. PLoS Curr. 2015;7:7. 10.1371/currents.outbreaks.0371243baa7f3810ba1279e30b96d3b6 26075141 PMC4455990

[24] Bundgaard H Bundgaard JS Raaschou-Pedersen DET von Buchwald C Todsen T Norsk JB Effectiveness of adding a mask recommendation to other public health measures to prevent SARS-CoV-2 infection in Danish mask wearers : a randomized controlled trial. Ann Intern Med. 2021;174(3):335-43. 10.7326/M20-6817 33205991 PMC7707213

