# Supplementary material for: Surveillance of SARS-CoV-2 infection based on self-administered swabs, Denmark, May to July 2022: evaluation of a pilot study
Source: Euro Surveill. 2023 Sep 21;28(38):2200907. doi: 10.2807/1560-7917.ES.2023.28.38.2200907 (PMC10515494; doi:10.2807/1560-7917.ES.2023.28.38.2200907)
Supplement: Supplement [file 22-00907_FOGH_SUPPLEMENT.pdf]

Supplementary Material

This supplementary material is hosted by Eurosurveillance as supporting information alongside the article "Surveillance of SARS-CoV-2 infection based on self-administered swabs, Denmark, May to July 2022: evaluation of a pilot study" on behalf of the authors who remain responsible for the accuracy and appropriateness of the content. The same standards for ethics, copyright, attributions and permissions as for the article apply. Eurosurveillance is not responsible for the maintenance of any links or email addresses provided therein.

Supplementary Table S1: ***Demographic characteristics of the tested blood donors and the 626 household members who also performed tests and submitted samples.***

	Tested blood donors	%	Tested household members	%
**N**	707	53	626	47
Female	437	61.8	291	46.5
Male	270	38.2	335	53.5
**Age group**				
0-4 years	0	0	14	2.2
5-9 years	0	0	49	7.8
10-14 years	0	0	87	13.9
15-19 years	4	0.6	57	9.1
20-29 years	96	13.6	61	9.7
30-39 years	81	11.5	37	5.9
40-49 years	139	19.7	76	12.1
50-59 years	220	31.1	129	20.6
60-69 years	159	22.5	96	15.3
70-79 years	8	1.1	19	3.0
80+ years	0	0	1	0.2
**Residence**				
Central Region	182	25.7	154	24.6
Region Zealand	96	13.6	100	16
Capital Region	199	28.1	179	28.6
Region North	70	9.9	53	8.5
Region South	160	22.6	140	22.4

Supplementary Figure S1*: A graphical representation of the sampling intervals during the study*

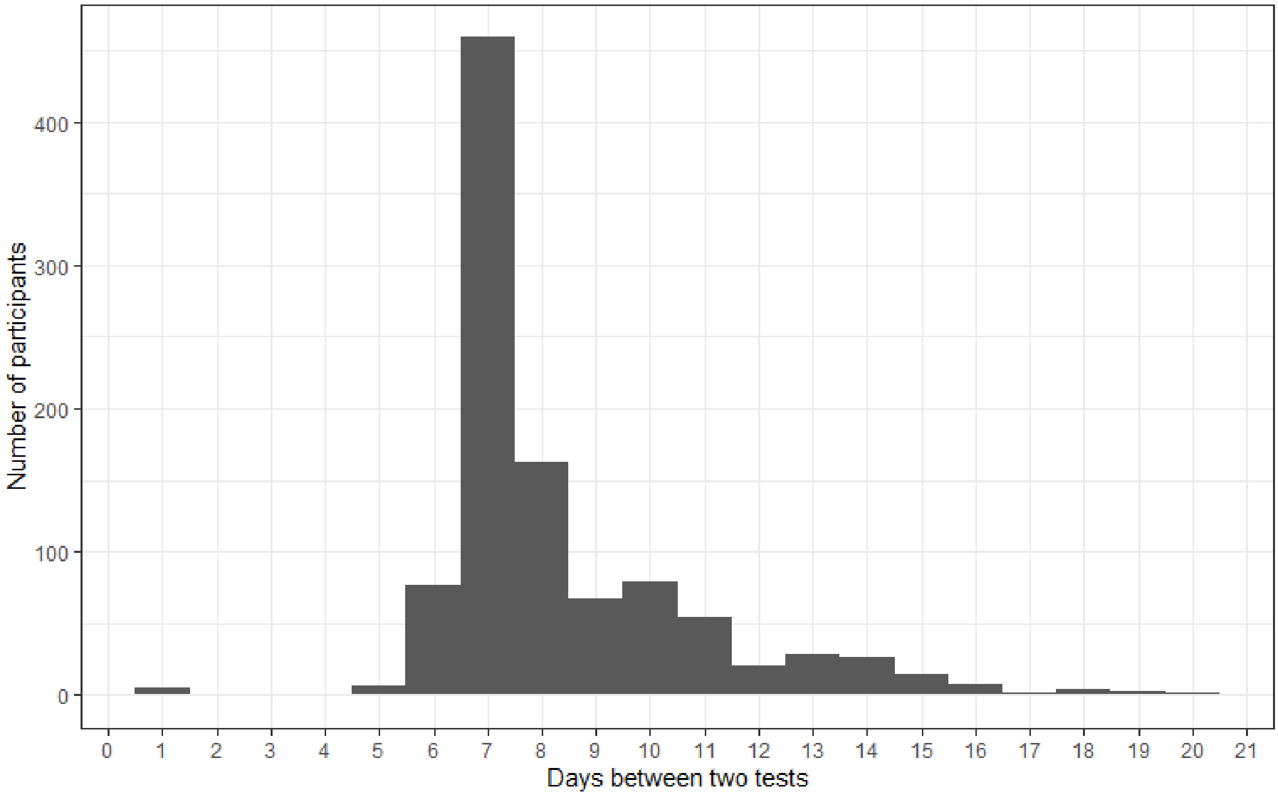

Supplementary Questionnaire S1: ***Used by participants to sign-up and/or explain their motivation for participating/not participating. Accessed through a link provided in the invitation letter sent via Digital Post.***

- What is your CPR number?
  - (DDMMYY-XXXX)
- Do you and/or some of your household members wish to participate in the "PCR Home Test Study"?
  - Yes
    - How many individuals, including yourself, will participate from your household (so we know how much testing material to send you)? Choose and option from the drop down menu.
      - (drop-down menu, response options: 1,2,3,4,5 or 6)
    - What is your/your family's motivation for participating? Choose an option:
      - There is a particularly vulnerable person in the family
      - I want to know if I or my family member has/gets Covid-19
      - I want to contribute to research/science
      - I want to contribute to the surveillance of infectious diseases like Covid-19
      - Other (Please specify your/your family's motivation)
        - (free text)
  - No
    - Indicate the reason that best explains why your household does not wish to participate in the "PCR Home Test Study": select one or more options
      - I do not want to take a swab myself/I cannot take a swab myself.
      - The process of sending the sample seems too complicated
      - I do not have a smartphone/I cannot figure out how to use apps/It seems too complicated to use an app
      - The response time for the test result is too long
      - Participation seems too time-consuming
      - I would like to, but I have travel or other plans that prevent me
      - I do not wish to participate in the surveillance of Covid-19
      - Other (Specify the reason for not wanting to participate)
        - (free text)

Supplementary Questionnaire S2: ***Answered in relation to every sampling. Answered on the web app.***

- Have you had symptoms of illness in the past week (7 days)?
    - No
    - Yes
        - Which symptoms? (select all that apply)
            - Fever
            - Muscle aches
            - Joint pains
            - Chest pain
            - Cough
            - Runny nose
            - Shortness of breath
            - Sore throat
            - Headache
            - Loss of smell
            - Loss of taste
            - Fatigue
            - Stomach pain and/or diarrhea
            - Nausea
            - Other
                - (Free text)
        - Have you been in contact with anyone you know was infected or later tested positive for COVID-19 in the past week?
        - No
        - Yes
            - With whom?
                - One or more household members were infected (or had been infected within the last 12 weeks) when we started the project
                - Family members in another household
                - Friends/acquaintances
                - Work colleague
                - School/daycare
                - Other
                    - (Free text)

Supplementary Questionnaire S3: ***Answered by participants after the study period, in order to assess experiences with participation. Accessed through a link provided in the "Thank you for participating" letter sent via Digital Post.***

- What is your CPR number?
    - (DDMMYY-XXXX)
    - Where did you swab from? Choose one of options below
        - Throat and then nose
        - Nose and then throat
        - Only nose
        - Only throat
        - Other
            - (free text)
- Have you completed and submitted all swabs?
    - Yes
    - No
        - What prevented you?
            - Swabbing was uncomfortable/awkward
            - Sending the sample was too complicated
            - There was a lack of swabbing material/the material broke I could not figure out how to use the app/it was too complicated to use an app
            - The app did not work
            - The response time for swabbing was too long
            - It has been too time-consuming to participate every week
            - I/we have been traveling or had other reasons that prevented me/us from participating
            - I/we did not want to participate in the Covid-19 surveillance after all
            - Other (Please specify what prevented you)
                - (free text)
    - The 4-week period was:
        - Too long
        - Could have been longer
        - Just right
        - Other
            - (free text)
    - Would your household be interested in participating if the "PCR Home Test Study" was rolled out again?
    - What length of participation period would be acceptable for your household? Choose one option
        - Yes, 1 month
        - Yes, 2 months
        - Yes, 2-6 months
        - Yes, over 6 months
        - No
